# Antimicrobial Activity and Mechanism of Inhibition of Silver Nanoparticles against Extreme Halophilic Archaea

**DOI:** 10.3389/fmicb.2016.01424

**Published:** 2016-09-13

**Authors:** Rebecca S. Thombre, Vinaya Shinde, Elvina Thaiparambil, Samruddhi Zende, Sourabh Mehta

**Affiliations:** ^1^Department of Biotechnology, Modern College of Arts, Science and CommercePune, India; ^2^National Center for Nanosciences and Nanotechnology, University of MumbaiMumbai, India

**Keywords:** silver nanoparticles, *Cinnamomum tamala*, extreme haloarchaea, antibiotic resistant, Baranyi model, antimicrobial, membrane permeability, cytotoxicity

## Abstract

Haloarchaea are salt-loving halophilic microorganisms that inhabit marine environments, sea water, salterns, and lakes. The resistance of haloarchaea to physical extremities that challenge organismic survival is ubiquitous. Metal and antibiotic resistance of haloarchaea has been on an upsurge due to the exposure of these organisms to metal sinks and drug resistance genes augmented in their natural habitats due to anthropogenic activities and environmental pollution. The efficacy of silver nanoparticles (SNPs) as a potent and broad spectrum inhibitory agent is known, however, there are no reports on the inhibitory activity of SNPs against haloarchaea. In the present study, we have investigated the antimicrobial potentials of SNPs synthesized using aqueous leaf extract of *Cinnamomum tamala* against antibiotic resistant haloarchaeal isolates *Haloferax prahovense* RR8, *Haloferax lucentense* RR15, *Haloarcula argentinensis* RR10 and *Haloarcula tradensis* RR13. The synthesized SNPs were characterized by UV-Vis spectroscopy, scanning electron microscopy, energy dispersive X-ray spectroscopy, dynamic light scattering, X-ray diffraction and Fourier transform infrared spectroscopy. The SNPs demonstrated potent antimicrobial activity against the haloarchaea with a minimum inhibitory concentration of 300–400 μg/ml. Growth kinetics of haloarchaea in the presence of SNPs was studied by employing the Baranyi mathematical model for microbial growth using the DMFit curve fitting program. The *C. tamala* SNPs also demonstrated cytotoxic activity against human lung adenocarcinoma epithelial cell line (A540) and human breast adenocarcinoma cell line (MCF-7). The mechanism of inhibition of haloarchaea by the SNPs was investigated. The plausible mechanism proposed is the alterations and disruption of haloarchaeal membrane permeability by turbulence, inhibition of respiratory dehydrogenases and lipid peroxidation causing cellular and DNA damage resulting in cell death.

## Introduction

Antibiotic resistance has been an emerging phenomenon in recent times due to the rampant use of antibiotics and spread of multi-drug resistance genes amongst microorganisms. An increase in the drug resistance genes has been observed in the resistome of environmental isolates due to the exacerbated use of antibiotics (Da Costa et al., 2013). The drug resistance has now disseminated from pathogenic bacteria to non-pathogenic environmental isolates due to the release of hospital effluents and sewage in marine habitats like the sea, estuaries, and aquatic systems (Vaidya, 2011; Da Costa et al., 2013). Extreme haloarchaea are halophilic archaea that occur in such marine and aquatic environments and thrive in the presence of 1.5–5 M NaCl (Oren, 2008; Thombre et al., 2016a). Their resistance to copious stress factors like oxygen limitation, salinity, temperature, and perchlorate is well documented (DasSarma, 2006; DasSarma et al., 2012). However, the phenomenon of antibiotic resistance in haloarchaea is less studied (Eckburg et al., 2003). The abundance of archaea in the human body is relatively lower as compared to that of bacteria, except for the members of the phylum Euryarchaeota (Aminov, 2013). Amongst archaea, methanogens like Methanobacteriales are known to colonize humans gut and cause periodontal disease (Mor et al., 2015). Haloarchaea are known to colonize human cells and till date, there are no reports on the pathogenicity of these organisms (Eckburg et al., 2003). As the pathogenic nature of some halophiles like *Vibrio* sp. and *Pseudomonas* sp. is known, the studies related to antibiotic resistance of halophilic archaea is imperative. Haloarchaea have been isolated from salted and fermented sea food (Roh et al., 2009; Lee et al., 2015). Hence it is essential to explore antimicrobial agents for inhibition and control of haloarchaea.

The antimicrobial applications of silver have been known since 1881 when the oligo dynamic properties of silver were utilized for prevention of eye infections (Russell and Hugo, 1994). Since then, silver has been heralded as an ideal metal for its antimicrobial potentials owing to its cost effectiveness, efficacy, and broad spectrum antimicrobial activities (Jain et al., 2009). The research in Nanotechnology has been increasing due to the application of nanomaterials in molecular sieving and separation, cancer therapy, pharmaceutics, and biotechnology (Jain et al., 2009; Joshi et al., 2014; McCarroll et al., 2014; Pyrgiotakis et al., 2015). Nanoparticles can be explored for their application in nanomedicine as nano-diagnostic platforms and biomarkers for diagnosis of pancreatic cancer (McCarroll et al., 2014). They are considered as efficient delivery agents for RNA interference (RNAi) inhibitors in RNAi based therapeutics (McCarroll et al., 2014). Engineered nanostructures have application in food safety and nanotoxicology for prevention and control of food borne pathogens (Pyrgiotakis et al., 2016). Silver nanoparticles (SNPs; size between 1 and 100 nm) have emerged as potent inhibitory agents due to their size, surface area, chemical properties, and antimicrobial potentials against microorganisms (Sondi and Salopek-Sondi, 2004; Jain et al., 2009; Mapara et al., 2015).

Classically, SNPs are synthesized using conventional top-down or bottom-up approach using physical or chemical techniques that may be costly, energy demanding and may involve chemicals that are toxic to the environment (Zinjarde, 2012). Biological methods of synthesis are hence emerging as efficacious approaches that utilize the abilities of actinomycetes (Ahmad et al., 2003), fungi (Bansal et al., 2005, 2011; Gade et al., 2008), bacteria (Parikh et al., 2011; Thombre et al., 2013), plant extracts (Shankar et al., 2003; Chandran et al., 2006; Ghosh et al., 2012; Thombre et al., 2014; Mapara et al., 2015) or yeast (Kowshik et al., 2003; Agnihotri et al., 2009; Apte et al., 2013) to reduce silver compounds to nanosilver. The synthesis of SNPs using plant based extracts is commonly known as ‘green synthesis’ is the most preferred method as it does not generate any toxic by-products, is safe and environment friendly (Bansal et al., 2011; Thombre et al., 2014).

In the past few years, there has been an upsurge in the reports on synthesis and antimicrobial activity of SNPs. SNPs are more toxic and potent than silver ions and this attribute has a cardinal role in its increased spectra of applications in biomedicine, antibacterial ointments, disinfectants, cancer therapeutics, and drug delivery (Duncan et al., 2004; Jain et al., 2009; Firdhouse and Lalitha, 2015). Nanoparticles are known to unfold proteins leading to inactivation of functional proteins via counterions in protein–nanoparticles interaction indicating its application in therapeutics (Ghosh et al., 2016). SNPs produced using the green synthesis methods are being investigated as potential antimicrobial and anticancer agents in biomedicine. The cytotoxic activity of biogenic SNPs against cancerous cell lines has been studied earlier. It is reported that the SNPs synthesized using extracts of *Piper longum*, *Melia dubia*, apple (*Malus domestica*) and Chaga mushroom (*Inonotus obliquus*) have anti-proliferative activity against human breast cancer cell line (Kathiravan et al., 2014; Lokina et al., 2014; Nagajyothi et al., 2014; Reddy et al., 2014). While the SNPs synthesized using *Tylophora indica* have cytotoxic activity against MCF-7 cell lines (Oke et al., 2015).

Bio-stabilized SNPs are known to be more toxic and potent against gram positive and bacteria and multidrug resistant (MDR) organisms and pathogens like *Mycobacterium tuberculosis* (Sarkar et al., 2015). SNPs synthesized using plant extracts have been reported to have antimicrobial activity against MDR and extensively drug resistant (XDR) *Pseudomonas* sp. (Mapara et al., 2015). Biogenic SNPs also enhance antibacterial activity of antimicrobial agents when used synergistically against drug resistant isolates of *Acinetobacter baumannii* (Ghosh et al., 2012).

However, the inhibitory activity of SNPs has not yet been explored on the microbial members of the metabolically diverse extremophiles belonging to the Domain Archaea. Some halophilic archaea have been isolated from fermented salted food products (Roh et al., 2009) and it is perused that they might have potential implications in human diseases (Eckburg et al., 2003; Aminov, 2013). It is thus imperative to screen the resistance of these organisms to antibiotics and explore for possible agents for inhibition of these antibiotic resistant organisms. Despite the accumulation of information in archaeal genomics, biochemistry, and biotechnological applications, little is known about the effect of metallic nanoparticles on these extremely resistant haloarchaea.

In the present study, bio-stabilized SNPs were synthesized using leaf extract of *Cinnamomum tamala* commonly known as bay leaf or Malabar leaf that is used for seasoning and culinary purposes in Asia. The plant is rich in phytochemicals and its active volatile compounds are known for its anticancer, antibacterial, anticonvulsant and antioxidant properties (Devi et al., 2007). The antimicrobial potential of *C. tamala* SNPs was assessed against four extremely resistant strains of halophilic archaea and the elucidation of the plausible inhibitory mechanisms of these SNPs was attempted. The current investigation is the first report on the antimicrobial effect of SNPs on haloarchaea and elucidation of the underlying inhibitory mechanism of SNPs against haloarchaea.

## Materials and Methods

### Ethical Approval

This article does not contain any studies conducted with human participants or animals performed by any of the authors.

### Preparation of Extract

The plant material used for the green synthesis of SNPs were dried leaves of *Cinnamomum tamala* known as Indian bay leaf or Malabar leaf commonly used as a spice. The dried spice leaves were obtained from local Indian stores and the sample was deposited at the Botanical Survey of India for authentication. The plant extract was prepared by adding 1 g of dried and thoroughly washed bay leaves powder in 100 ml of sterile deionized water in a sterile Erlenmeyer flask and boiled for 20 min (Francis et al., 2014). After boiling, the bay leaf extract was cooled and filtered through Whatman no. 1 filter paper and stored at 4°C in the dark till further use.

### Synthesis of SNP

The bio-stabilized SNPs were synthesized by the method described by Thombre et al. (2014). The reduction of silver to nanosilver was obtained by addition of 10 ml of the bay leaf extract to 90 ml of 100 mM AgNO_3_ (Sigma Aldrich, Germany) and incubated at 37°C till color change from pale yellow to dark brown was observed. After color change was achieved, the SNPs in the reaction mixture were subjected to centrifugation at 10,000 × *g* for 30 min and the obtained pellet was resuspended in sterile deionized water and washed repeatedly to remove impurities. The SNPs obtained were dried and stored in a cool dark place till further use.

### Characterization of SNP

The surface plasmon resonance of the biosynthesized SNP’s was characterized by observation of the spectra using a UV-Vis spectrophotometer (UV-2450, Shimadzu, Japan). The phase formation and crystalline nature of the SNPs was ascertained by X-ray Diffraction (XRD) analysis using an X-Ray diffractometer (D8 ADVANCE, Bruker, Germany). The ionic composition of the SNP was studied by energy-dispersive X-ray spectroscopy (EDS) using Scanning electron microscope (JSM-7600F, Jeol). The morphology was studied using a Field Emission Gun-Scanning Electron Microscope (FEG-SEM; Inspect-50, FEI, USA). The presence of plant peptides that may have coated and bio stabilized the SNP’s were detected by Fourier transform infrared spectroscopy (FT-IR) using 3000 Hyperion Microscope with Vertex 80 FTIR system (Bruker, Germany). The zeta potential value (ζ values) is used to assess the particle stability of nanoparticles due to electrostatic repulsion. The zeta-potential of the SNP’s was studied using a dynamic light scattering instrument (Nano ZS-90, Malvern instruments, UK).

### Extreme Haloarchaeal Isolates and Culture Conditions

The extreme haloarchaea used in this study were isolated in our laboratory previously from the thalossohaline salterns of Mumbai, India and deposited in Microbial Culture Collection (MCC), National Centre for Cell Science, Pune, India (Thombre et al., 2016b). The isolates used were *Haloarcula tradensis* strain RR13 (GenBank/EMBL/DDBJ accession number KP712894, MCC 2922), *Haloarcula argentinensis* strain RR10 (GenBank/EMBL/DDBJ accession number KP712898, MCC 2923), *Haloferax prahovense* strain RR8 (GenBank/EMBL/DDBJ accession number KP712893, MCC 2957) and *Haloferax lucentense* strain RR15 (GenBank/EMBL/DDBJ accession number KP712896, MCC 2924). The medium used for growth of the haloarchaea was Sehgal and Gibbons (SG) medium containing (g/L) casamino acids (7.5), yeast extract (10), potassium chloride (2), trisodium citrate (3), magnesium sulfate (20) and pH- 7.2 supplemented with 4.28 mol l^-1^ sodium chloride (Sehgal and Gibbons, 1960). The sensitivity of the haloarchaea to antibiotics was assessed by disk diffusion method as per Clinical and Laboratory Standards Institute [CLSI] (2012) guidelines. Briefly, the inoculum (absorbance corresponding to 0.5 McFarland standard) was spread on SG agar medium and antibiotic disks (Himedia, Mumbai, India) were placed aseptically on it. The plates were incubated at 37–40°C for 5–7 days and the diameters of zones of inhibition obtained were measured. Currently the data regarding the standard guidelines prescribed by CLSI for interpretive criteria and break points for antibiotic sensitivity testing and drug resistance in haloarchaea are meager. Hence, the results obtained were compared with interpretive criteria for gram negative bacteria and *Enterobacteriaceae* and interpreted according to the CLSI guidelines for Gram-negative bacteria (Clinical and Laboratory Standards Institute [CLSI], 2014).

### Growth Kinetic Studies of Haloarchaea in Presence of SNPs Using Baranyi Mathematical Model

The MIC of SNPs on haloarchaea was determined by agar dilution method as described previously (Popescu and Dumitru, 2009). The kinetics of microbial growth of the haloarchaea were studied in the presence of SNPs using concentrations below the Minimal Inhibitory Concentration (MIC) using the Baranyi model (Baranyi and Roberts, 1994). For studying the growth curve, the balanced growth of haloarchaeal isolates was obtained (Robinson et al., 2005) and the cultures *Hal. argentinensis* RR10, *Hal. tradensis* RR13, *Hfx. prahovense* RR8 and *Hfx. lucentense* strain RR15 were inoculated in 100 ml SG broth supplemented with 4.28 mol l^-1^ sodium chloride and incubated in an orbital shaker at 40°C and 100 rev min^-1^. The growth was monitored by measuring the absorbance at 600 nm every 24 h using UV-Vis spectrophotometer (UV-2450 Shimadzu, Japan). The lag phase was calculated by fitting the growth curve plot of concentration of cells versus –time with the Baranyi model (Baranyi and Roberts, 1994) using the curve-fitting DMFit program (Metris et al., 2006; Salgaonkar et al., 2016). The generation time (*g*) and the specific growth rate constant (*k*) was calculated from the growth curve as described by Robinson et al. (2005).

### Effect of SNP on Membrane Leakage of Reducing Sugars and Proteins in Haloarchaea

The effect of SNPs on membrane leakage of reducing sugars and proteins released from the intracellular cytosol of the cells after treatment of SNPs was studied by modification of the method described by Li et al. (2010) to suit the growth of haloarchaea. In all the experiments, the inoculum culture was prepared in SG medium containing 4.28 mol l^-1^ sodium chloride to prevent the lysis of haloarchaeal cell membrane due to lowering of NaCl content. The haloarchaea, *Hal. argentinensis* RR10, *Hal. tradensis* RR13, *Hfx. prahovense* RR8, and *Hfx. lucentense* strain RR15 were inoculated in 10 ml SG broth containing 300 μg/ml SNPs to obtain a final cell density of 10^8^ cells/ml. The haloarchaeal cultures were incubated at 40°C and 100 rev min^-1^ in an orbital shaker. After 24 h, aliquots were withdrawn from the culture medium and centrifuged at 10,000 × *g* for 30 min at 4°C. The supernatant obtained was immediately stored at -20°C. The reducing sugar in the supernatant was estimated as described by Miller (1959) and the proteins in the supernatant were estimated by Bradford (1976) method.

### Effect of SNP on Respiratory Chain Dehydrogenase Activity

The respiratory chain dehydrogenase activity of haloarchaea was evaluated by spectrophotometric assay based on the reduction of iodonitrotetrazolium chloride (INT) by the haloarchaeal respiratory chain dehydrogenases. The haloarchaeal cells (10^8^ cells/ml) were inoculated in SG medium containing 300 μg/ml SNP and incubated at 40°C and 100 rev min^-1^ in an orbital shaker. After incubation, the culture medium was centrifuged at 10,000 × *g* for 30 min at 4°C and the cell pellet was washed with sterile SG medium containing 4.28 mol l^-1^ sodium chloride. The cell pellet was resuspended in sterile phosphate buffered saline (900 μl) supplemented with 4.28 mol l^-1^ sodium chloride and 0.5% INT (100 μl) was added in the reaction mixture. The reaction mixture was incubated in the dark at 40°C for 2 h. The dehydrogenase activity was further estimated and measured spectrophotometrically at 490 nm as described earlier (Li et al., 2010).

### Effect of SNP on Membrane Lipid Peroxidation in Haloarchaea

Oxidative stress causes the formation of unstable lipid peroxides in microbial cells that decompose to form reactive compounds like malondialdehyde (MDA) and this process of lipid peroxidation causes cellular damage. Lipid peroxidation can be detected by the thiobarbituric acid-reactive substance (TBARS) assay in which MDA forms a complex with thiobarbituric acid (TBA) that can be quantified spectrophotometrically (Joshi et al., 2011). For the TBARS assay, *Hal. argentinensis* RR10, *Hal. tradensis* RR13, *Hfx. prahovense* RR8, and *Hfx. lucentense* strain RR15 were inoculated in SG broth containing 0 and 300 μg/ml SNP to obtain a final cell density of 10^8^ cells/ml. The haloarchaeal cultures were incubated at 40°C and 100 rev min^-1^ in an orbital shaker. After 24 h, culture medium was centrifuged at 10,000 × *g* for 30 min at 4°C. The cell pellet was washed and re-dispersed in 10% SDS (500 μl) to which 2.5 ml TBA buffer was added. The reaction mixture was incubated at 95°C for 60 min and cooled to 25°C. Thereafter, the reaction mixture was centrifuged at 5000 × *g* for 15 min to remove cell debris. The absorbance of the supernatant was measured at 532 nm using UV-Vis spectrophotometer (UV-2450 Shimadzu, Japan) and the membrane lipid peroxidation was quantified using standard curve of MDA as described by Hong et al. (2012).

### Antimicrobial Activity of SNPs

Antimicrobial activity of SNPs was evaluated against representative bacteria as described by Thombre et al. (2012). The inoculum (100 μl; adjusted to 0.5 McFarland standard) was spread on Mueller Hinton Agar (MHA) for bacteria and SG agar for haloarchaea to obtain a lawn of confluent growth. Wells of 6 mm diameter were made in the agar using a gel puncture and ∼20 μl SNPs was added aseptically in the wells and the plates were incubated at 37°C for 24 h for bacteria and 37–40°C for 72–96 h for haloarchaea. The concentrations of SNPs used for the antibacterial study were based on the MIC values of each organism. The microbial strains used in the present study were the haloarchaea RR8, RR10, RR13, and RR15 as mentioned above and laboratory isolates *Escherichia coli*, *Staphylococcus aureus, Bacillus subtilis*, and *Pseudomonas* sp. as described earlier (Thombre et al., 2012). The formation of clear zone around the well depicts antimicrobial activity and the diameter of zones of inhibition were measured.

### Cytotoxic Activity of SNPs

The cytotoxic activity of biosynthesized SNPs against human lung adenocarcinoma epithelial cell line (A540) and on human breast adenocarcinoma cell line (MCF-7) was tested by standard method as described by Thombre et al. (2013). Briefly, the cell lines were seeded in 96 well microtitre plate in DMEM medium with 10% Fetal Calf Serum to obtain a density of 1 × 10^4^ cells/well. The cells were treated with 100–1000 μg/mL biosynthesized SNPs and incubated at 37°C for 24 h in a 5% CO_2_ humidified incubator (Thermo Scientific, USA). After 24 h, 20 μl of MTT (50 μg/ml) was added in each well and incubated for about 4–6 h in dark at 37°C. After incubation with MTT, the microtitre plates were centrifuged (1000 × *g* for 10 min), and 150 μl of dimethyl sulfoxide was added in each well and incubated on an orbital shaker at 37°C for 10 min at 90 rev min^-1^. The absorbance of the color developed was recorded at 492 nm on micro plate reader (imark^TM^, Bio-Rad, USA) and the cytotoxicity was calculated as described earlier (Thombre et al., 2013). Suitable blanks and positive controls were maintained and the assays was performed in triplicate and repeated twice. Cytotoxicity data were reported as the mean ± SE of six measurements and IC_50_ values were compared by paired *t*-test (*p* < 0.05 was considered significant).

### Statistical Analysis

All experiments were performed in triplicates and repeated twice for obtaining statistically significant values. All values were expressed as the mean ± standard error (SE). Statistical analysis was performed using Microsoft Excel 2016. Statistical significance was calculated within groups using the *t*-test and the value of *P* < 0.05 was considered to be statistically significant.

## Results

### Synthesis and Characterization of SNP

**FIGURE 1:**
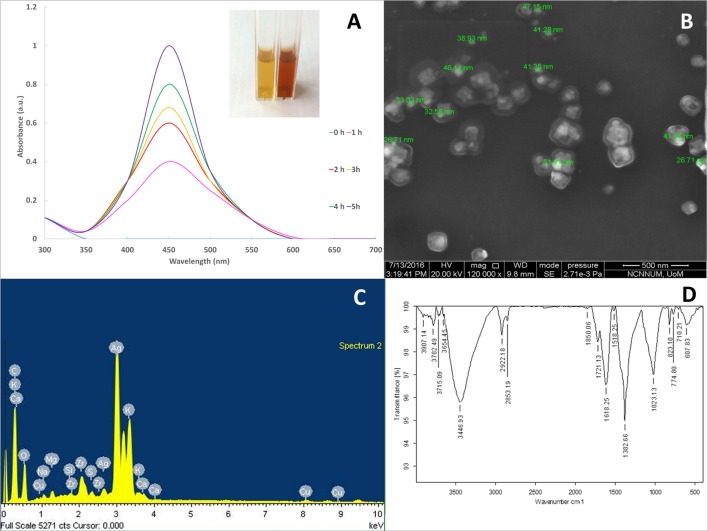
**(A)** UV-Vis spectra of SNP synthesized using *C. tamala* extract; inset figure: color change from pale yellow (0 h) to dark brown (5 h) indicating synthesis of nanoparticles. **(B)** FEG-SEM of SNPs synthesized using *C. tamala* leaf extract depicting spherical nanoparticles. **(C)** Energy Dispersive X-ray spectrum of SNPs biosynthesized using *C. tamala* leaf extract. **(D)** Fourier transform infrared spectra of SNPs synthesized using *C. tamala* leaf extract.

The plant material was authenticated as *Cinnamomum tamala* (Buch. -Ham.) T. Nees & Eberm. (Ref. No. BSI/WRC/IDEN.CER/2016/79) belonging to the family *Lauraceae*. The biosynthesis of SNPs was performed by using leaf extract of *C. tamala* and the color change in the reaction mixture from pale to dark brown indicative of the bioreduction of silver nitrate was observed at regular time intervals (1 h) using UV-Vis spectrophotometry (**Figure 1A**). The absorption maxima was obtained at 450 nm due to the surface plasmon excitations indicating the presence of SNPs (Sun et al., 2001; Jae and Beom, 2009). The biosynthesized SNPs were further characterized for its morphology by SEM analysis. The analysis revealed them to be spherical in shape with an average size ranging from ∼25–50 nm (**Figure 1B**) and the EDS confirmed the presence of silver in ionic composition of the SNP (**Figure 1C**). The *C. tamala* SNPs had a negative zeta potential of -27.3 mv (**Supplementary Figure S1**).

During biosynthesis the nanoparticles tend to form nuclei and seeds. These seeded nuclei can become single crystals or demonstrate single/multiple twinning and depending on the stacking as well as twin boundary effect, they lower their energy and become trapped in fixed morphologies that may have different facets [(100), (111), (110) facets] (Sajanlal et al., 2011). The crystalline nature of the SNPs was reconfirmed by the XRD analysis (**Supplementary Figure S2**). The peaks were observed between 20 and 80° and the Braggs reflections show presence of (111), (200), (220), and (311) planes that demonstrate face-centered-cubic structure (FCC) for nanosilver denoting complete bio-reduction of silver nitrate obtained by the plant extract and are in agreement with existing data (JCPDS file no. 04-0783; Thombre et al., 2014; Annamalai and Nallamuthu, 2016). The other peaks may be due to bioorganic-phases on SNP surface and the XRD results clearly demonstrate crystalline nature of SNPs (Annamalai and Nallamuthu, 2016). The average particle size was evaluated using the Debye–Scherrer’s formula and it was found to be in the range of the particle size as detected in SEM (John and Florence, 2009).

The SNPs tend to agglomerate and their agglomeration is restricted due to capping or stabilizing agents like polyols (Wiley et al., 2007) which may be present in plant extract. The presence of plant origin molecules present on the SNPs were detected using FTIR measurements (**Figure 1D**). The typical stretching and peaks observed between 500 to 1850 cm^-1^ and 2800 to 3900 cm^-1^ indicate the presence of N-H stretch of carbonyl amine, C-N stretch of aliphatic amines and amide linkages between proteins indicating the presence of peptides associated with the nanoparticles (Bansal et al., 2005; Huang et al., 2007). It is known that plant peptides and proteins play a role in stabilization of SNPs due to their affinity to metals and the binding of free amine and/or cysteine residues of the peptides to the SNPs thereby stabilizing them (Gole et al., 2001; Lin et al., 2005).

### Antibiotic Resistance of Haloarchaea

The phenomenon of drug resistance is extensively studied in bacteria. Bacteria are considered drug resistant when they exhibit their resistance to drugs encompassing different classes of antibiotics like cephalosporins, aminoglycosides, carbapenems, penicillin, polymyxins, β-lactamase inhibitors, quinolones, fluoroquinolones, phosphonic acids and polymyxins (Magiorakos et al., 2012; Mapara et al., 2015). Currently the reports related to drug resistance in haloarchaea are scare (Eckburg et al., 2003). The resistance of haloarchaea to antibiotics may be attributed to lack of peptidoglycan (conferring resistance to ß-lactums), lack of target sites for antibiotics, unique archaeal cell wall structure and metabolism (Dridi et al., 2011; Khelaifia and Drancourt, 2012). The antibiotic resistance of haloarchaea was assessed using disk diffusion method as prescribed by Clinical and Laboratory Standards Institute [CLSI] (2012) and the results are presented in **Table 1**. The haloarchaea used in the current study were resistant to nalidixic acid, streptomycin, gentamicin, tetracycline, erythromycin, chloramphenicol, cephalothin, and clindamycin.

**Table 1:** Antibiotic resistance profile of haloarchaea by disk diffusion method (Isolates: *Haloferax prahovense* RR8, *Haloferax lucentense* RR15, *Haloarcula argentinensis* RR10, and *Haloarcula tradensis* RR13).

Name of antibiotic	Concentration (μg/ml)	Class	Interpretive Criteria for Zone Diameter^∗^ (mm)			Antibiotic resistance profile of haloarchaea			
				
			S	I	R	RR8	RR15	RR10	RR13
Ampicillin	10	β-Lactam	≥17	14–16	≤13	R	R	R	R
Nalidixic acid	30	Quinolone	≥19	14–18	≤13	R	R	R	R
Streptomycin	25	Aminoglycoside	≥15	12–14	≤11	R	R	R	R
Gentamicin	10	Aminoglycoside	≥15	13–14	≤12	R	R	R	R
Bacitracin	10	Polypeptide	–	–	–	S	S	R	S
Novobiocin	30	Aminocoumarin	–	–	–	S	S	S	S
Ciprofloxacin	5	Fluoroquinolone	≥21	16–20	≤15	S	S	S	S
Tetracycline	30	Tetracycline	≥15	12–14	≤11	R	R	R	R
Erythromycin	15	Macrolides	≥23	14–22	≤13	R	R	R	R
Chloramphenicol	30	Phenicol	≥18	13–17	≤12	R	R	R	R
Cephalothin	30	Cephalosporin	≥18	15–17	≤14	R	R	R	R
Clindamycin	2	Lincosamide	≥21	15–29	≤14	R	R	R	R
Trimethoprim	25	Folate pathway inhibitor	≥16	11–15	≤10	S	S	S	S


*R, resistant; I, intermediate; S, sensitive; nd, not determined; Interpretive criteria of *^∗^Enterobacteriaceae* or *Gram-negative bacteria* as per guidelines of CLSI M100-S24 for disk diffusion.*

### Determination of Minimal Inhibitory Concentration and Antibacterial Activity of SNP

The minimum inhibitory concentration of SNPs against haloarchaea was evaluated and the inhibitory activity of SNPs against haloarchaea and bacteria was assessed (**Table 2**). All the haloarchaeal strains RR8, RR13 and RR15 demonstrated an MIC of 300 μg/ml SNP except *Hal. argentinensis* RR10 whose MIC was 400 μg/ml SNP. The haloarchaea used in the present study are extremely resistant and require 20–30% NaCl for growth and are capable of surviving in the presence of metals like manganese (upto 500 mM), lithium (300 mM), magnesium (500 mM), and chemicals like perchlorate (upto 500 mM). Besides all the haloarchaeal strains used in the current investigation are resistant to multiple antibiotics (**Table 1**). The ability of the SNPs to inhibit the extremely resistant haloarchaea was significant as the haloarchaea used in the present study are polyextremophiles. *Haloferax* sp. was more sensitive to SNPs than *Haloarcula* sp. Amongst *Haloarcula, Hal. tradensis* was more sensitive to the SNPs than *Hal. argentinensis*. A comparison of the toxicity of SNPs between bacteria and haloarchaea was done and it was found that the SNPs showed significant inhibition in both the groups (**Table 2**). The toxicity of SNPs in bacteria was higher. The toxicity of silver and nanosilver is known against many organisms. The antimicrobial potentials of SNPs are dependent on size, surface charges and capping agents that stabilize the nanoparticles (Sarkar et al., 2015). SNPs have demonstrated antimicrobial activity against *Escherichia coli, Bacillus* sp*., Klebsiella sp., Staphylococcus aureus* (Thombre et al., 2012), *Enterococcus faecium* (Theophel et al., 2014), *Mycobacterium tuberculosis* (Sarkar et al., 2015), and XDR clinical isolates of *Pseudomonas aeruginosa* (Mapara et al., 2015). However, this is presumably the first report on antimicrobial activity of SNPs against extremely resistant halophilic archaea.

**Table 2:** Inhibitory effect of SNPs synthesized using *C. tamala* extract against haloarchaea and bacteria.

No.	Test Organism	Zone of inhibition (mm)
1	*Haloferax lucentense*	10.5
2	*Haloferax prahovense*	11.0
3	*Haloarcula tradensis*	8.5
4	*Haloarcula argentinensis*	7.5
5	*Escherichia coli*	10.0
6	*Staphylococcus aureus*	11.0
7	*Bacillus subtilis*	11.0
8	*Pseudomonas* sp.	8.0


### Growth Kinetic Studies of Haloarchaea in Presence of SNP

**FIGURE 2:**
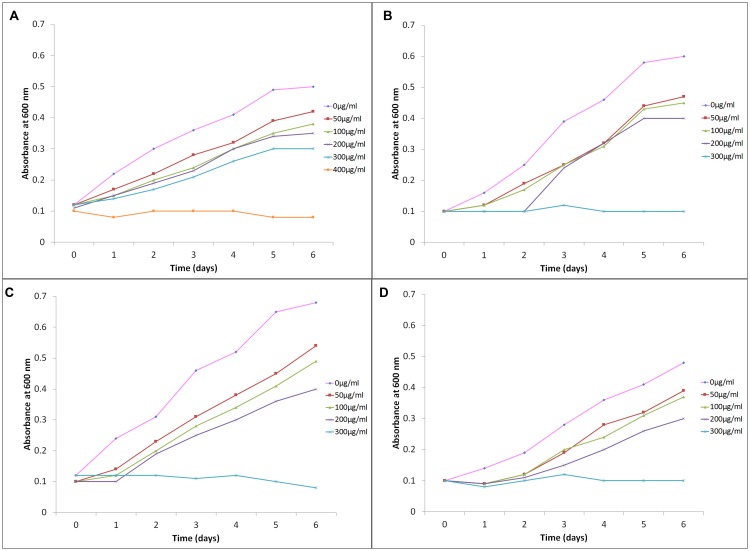
**Growth curve of Haloarchaea in different concentrations of SNPs: **(A)***Haloarcula argentinensis*, **(B)***Haloarcula tradensis*, **(C)***Haloferax lucentense*, and **(D)***Haloferax prahovense***.

The growth kinetics of haloarchaea in the presence of varying concentrations of SNPs was studied in SG medium. The inhibitory activity of SNPs in haloarchaea was dose dependent and the growth curves are depicted in **Figure 2**. The control (devoid of SNPs) of all the four cultures showed maximum growth in absence of SNPs. When the concentration of SNPs increased, the growth was lower as evidenced by the absorbance. *Hfx. prahovense* was the most susceptible to SNPs. The Baranyi model (using the DMFit curve fitting program) was employed for calculation of the lag phase induced in haloarchaeal population due to the stress caused by the presence of SNPs in the medium. This model is a mathematical model where a factor (α_0_) is used to describe the physiological state of the cell during transitioning from lag to exponential phase (Baranyi and Roberts, 1994). A longer lag is attributed to stress produced by extrinsic parameters which is the presence of nanoparticles in the growth medium in the present study. Baranyi model is the most common stochastic model used to accurately predict the lag time of cells in a given microbial population. A distinct lag phase (48 ± 2 h) was observed when *Hal. tradensis* RR13, *Hfx. prahovense* RR8 and *Hfx. lucentense* strain RR15 were exposed to 200 μg/ml SNP (Lesser than MIC; **Table 3**). The growth kinetics of the haloarchaea in the presence of varying concentrations of SNPs is presented in **Table 3**. No distinct lag phase was observed when *Hal. argentinensis* RR10 was exposed to SNPs, however, the SNPs retarded the growth of *Hal. argentinensis* as observed in change of cellular doubling time from 16 to 32 h (**Table 3**). The generation time of all the other isolates was affected due to the presence of SNPs in the medium and the generation time ranged between 20 and 28 h for all the other haloarchaea. The kinetic studies confirm that the SNPs have a dose and time dependent effect on growth of the extremely resistant haloarchaea.

**Table 3:** Growth kinetics of the haloarchaea in the presence of varying concentrations of SNPs.

Organism	Concentration of SNP (mM)	λ (h)	*g* (h)	μ (h^-1^)	*k*
*Haloarcula argentinensis*	0	0	16	0.074622	0.0433
	100	0	26	0.045714	0.0266
	200	0	28	0.043214	0.02475
	300	0	32	0.048857	0.0216
*Haloarcula tradensis*	0	0	17	0.091071	0.04076
	100	12	22	0.064643	0.0315
	200	48	20	0.115124	0.03465
	300	–	–	–	–
*Haloferax lucentense*	0	0	17	0.096786	0.0407
	100	22	24	0.072588	0.0288
	200	24	24	0.054643	0.0288
	300	–	–	–	–
*Haloferax prahovense*	0	0	20	0.066071	0.0346
	100	12	24	0.061918	0.0288
	200	24	28	0.051767	0.02475
	300	–	–		–


*λ, lag phase time; *g*, doubling time; μ, maximum specific growth rate; *k*, growth rate constant.*

### Effect of SNP on Membrane Leakage of Reducing Sugars and Proteins in Haloarchaea

**FIGURE 3:**
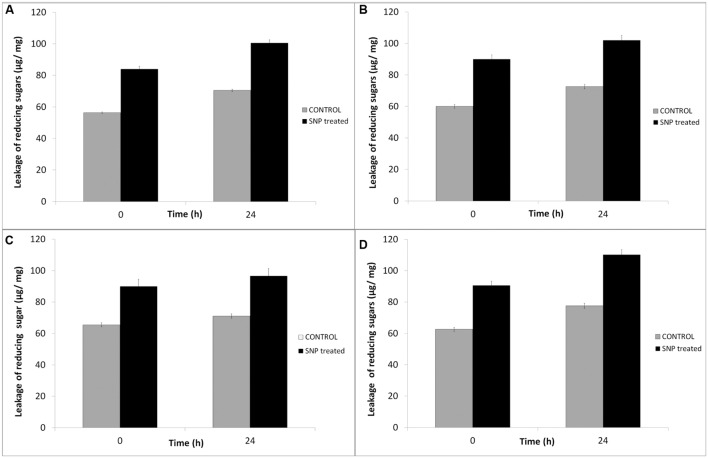
**Effect of SNPs on membrane leakage of reducing sugars in haloarchaea **(A)***Haloarcula argentinensis*, **(B)***Haloarcula tradensis*, **(C)***Haloferax lucentense*, and **(D)***Haloferax prahovense.*** Error bar represents standard error.

**FIGURE 4:**
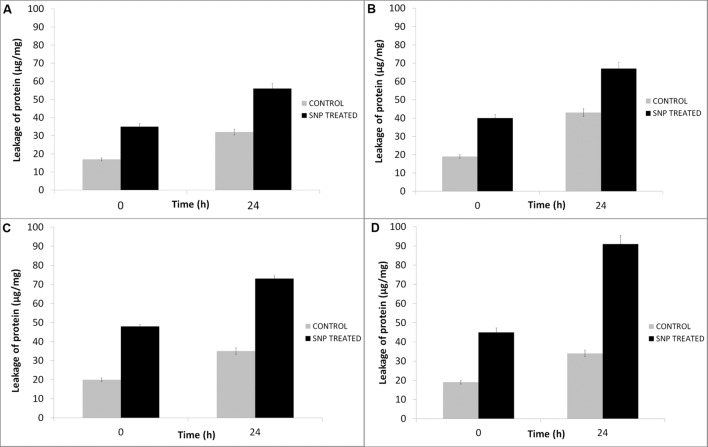
**Effect of SNPs on membrane leakage of proteins in haloarchaea **(A)***Haloarcula argentinensis*, **(B)***Haloarcula tradensis*, **(C)***Haloferax lucentense*, and **(D)***Haloferax prahovense*.** Error bar represents standard error.

The effect of SNPs on membrane leakage of reducing sugars was studied and is presented in **Figure 3**. The membrane leakage was almost negligible at time 0. After treatment with SNPs, the leakage of sugars was observed in all the haloarchaeal strains. The leakage was maximum in *Hfx. prahovense* and minimum in *Hfx. lucentense.* Similarly, the amount of proteins leaked from the membranes damaged after treatment of SNP was studied. It was observed that protein leakage was significantly higher in haloarchaeal samples treated with SNPs after 24 h (**Figure 4**). The control demonstrated lesser protein leakage as compared to the SNP treated group. The protein leakage was maximum for *Hfx. prahovense* with 2.67 times greater protein leakage as compared to control cells. For other haloarchaea, the magnitude of leakage in terms of fold increase as compared to control cells was *Hfx. lucentense* (2.08) > *Hal. argentinensis* (1.7) > *Hal. tradensis* (1.55). The results indicate that the SNPs affect the cell membrane integrity causing leakage of reducing sugars and proteins from the intracellular cytosol. Despite the fact that haloarchaeal membranes are considered to be very robust, they still fail to maintain their membrane integrity on exposure to SNPs.

### Effect of SNP on Respiratory Chain Dehydrogenase Activity

**FIGURE 5:**
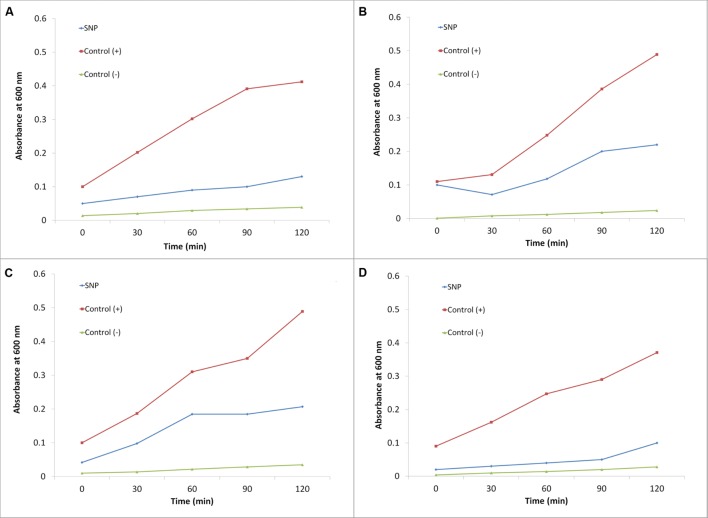
**Effect of SNPs on respiratory chain dehydrogenase activity in haloarchaea **(A)***Haloarcula argentinensis*, **(B)***Haloarcula tradensis*, **(C)***Haloferax lucentense*, and **(D)***Haloferax prahovense***.

The effect of SNPs on respiratory chain dehydrogenases of haloarchaea is depicted in **Figure 5**. It is observed from the results that the control cells consisting of haloarchaea incubated without the SNPs demonstrated increased respiratory dehydrogenase activity. The activities of the negative control consisting of heat inactivated cells were almost negligible. The respiratory dehydrogenase activity of *Hal. tradensis* and *Hfx. lucentense* decreased with time. The activity of respiratory chain dehydrogenase of *Hal. argentinensis* and *Hfx. prahovense* continued to increase with time.

### Effect of SNP on Membrane Lipid Peroxidation in Haloarchaea

**FIGURE 6:**
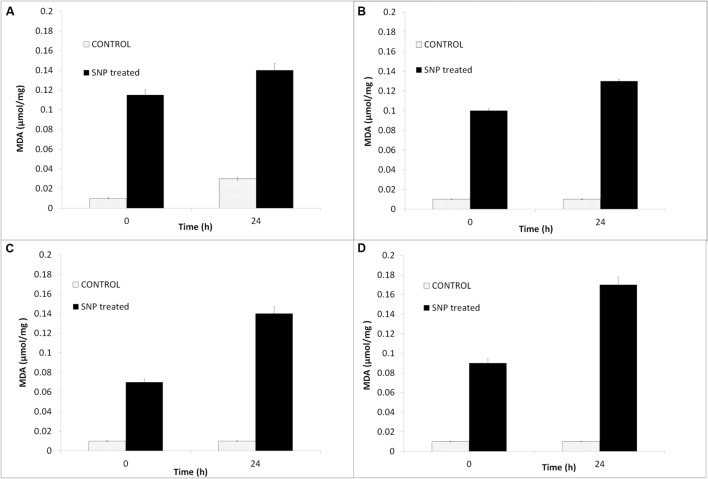
**Effect of SNPs on membrane lipid peroxidation as detected by MDA content in haloarchaea **(A)***Haloarcula argentinensis*, **(B)***Haloarcula tradensis*, **(C)***Haloferax lucentense*, and **(D)***Haloferax prahovense.*** Error bar represents standard error.

The damage caused by SNP causes lipid peroxidation in haloarchaea which was detected by estimation of the MDA. It is observed from **Figure 6** that the MDA content was increased in the haloarchaeal cells treated with SNPs. Maximum MDA content was observed in *Hfx. prahovense* followed by *Hfx. lucentense*. Amongst the haloarchaea, MDA content was lesser in both the *Haloarcula* sp.

### Cytotoxic Activity of SNPs

The cytotoxic effect of SNPs synthesized using *C. tamala* extract was studied on two different cell lines, human lung adenocarcinoma epithelial cell line (A540) and human breast adenocarcinoma cell line (MCF-7) using MTT assay. The IC-50 value for the *C. tamala* SNPs against A540 and MCF-7 cell lines were 50 μg/ml and 100 μg/ml, respectively. The cytotoxic activity of SNPs produced by using plant extract has been studied extensively. The phytoconstituents and chemical properties of the plant material play a significant role in the cytotoxic activity of the SNPs. The plant material *C. tamala* used in the present study was investigated for the presence of phytochemicals qualitatively and the flavonoids and phenolics were estimated quantitatively as described by Devi et al. (2007). The aqueous plant extract is rich in flavonoids, phenolics, antioxidants and tannins (**Supplementary Table S1**) and these phytochemicals may also play a crucial role in cytotoxicity. The *C. tamala* plant extract is non-toxic to normal Human Peripheral Blood Mononuclear Cells (PBMC) and does not have any *in vitro* haemolytic activity against human blood cells as reported earlier (Thanekar et al., 2013) indicating its non-toxic nature to human cells.

## Discussion

Extreme haloarchaea are a metabolically diverse group of prokaryotic archaeabacteria belonging to the family *Halobacteriaceae* that require minimum 1.5 to 5 M NaCl for growth and survival (Kushner, 1978; Oren, 1994, 2012; Grant et al., 2001). Almost all haloarchaeal members of the *Halobacteriaceae* family produce red-pink pigments except *Natrialba* sp. (Amoozegar et al., 2012). The isolates used in the present study belong to the genus *Haloarcula* and *Haloferax* proposed by Torreblanca et al. (1986). The cell membrane of these haloarchaea is characterized by the absence of peptidoglycan and murein and the presence of S-layers made of glycoproteins and negatively charged amino acids stabilized by sodium and other divalent ions (LoBasso et al., 2008). The main phospholipids present in haloarchaeal membranes are phosphatidylglycerol (PG), phosphatidylglycerosulfate (PGS), phosphatidylglycerophosphate methyl ester (PGP-Me), archaeal cardiolipin (bisphosphatidylglycerol, BPG) and neutral lipids like squalene, carotene, vitamin MK-8, and retinal isomers (LoBasso et al., 2008; Amoozegar et al., 2012). The membrane structure and cellular metabolism play a pivotal role in enhancing resistance of haloarchaea to harsh environments and stressful growth parameters like extreme salinity, temperature, perchlorate, and oxygen limitation (DasSarma et al., 2012; Chitnis and Thombre, 2014; Thombre and Oke, 2015; Thombre et al., 2016b). In order to survive in such stresses, they operate manifold strategies of adaptation like the ‘salt-in’ strategy (Oren, 2008), ‘organic osmolytes strategy’ (Galinski, 1995; Da Costa et al., 1998), production of stress proteins (Chitnis and Thombre, 2014), production of red-orange carotenoid pigments like bacterioruberin (Thombre et al., 2016b) and employment of other cellular concerted mechanisms.

In addition to biotic and abiotic stress, antibiotic resistance is a common phenomenon in archaea (Dridi et al., 2011). However, there are no reports on the extensive screening of haloarchaea to antibiotics used for treatment of infections in humans. The resistance of haloarchaea to antibiotics is studied as a part of biochemical characterization of the isolates and studies detailing the MIC of antibiotics using standard broth/agar dilution methods are rare. In the present study, we studied the resistance of haloarchaea to the antibiotics commonly used to treat bacterial infections using CLSI guidelines prescribed for the selection and dosage of antibiotics against gram negative bacteria. The antibiotics chosen for screening of haloarchaeal resistance belonged to β-Lactam, Quinolone, Fluoroquinolone, Aminoglycoside, Macrolides, Phenicol, Cephalosporin, Folate pathway inhibitor, and Lincosamide groups. The haloarchaea used in the present study were sensitive to Bacitracin, Novobiocin, Ciprofloxacin, and Trimethoprim and were resistant to Nalidixic acid (Quinolone), Streptomycin (Aminoglycoside), Gentamicin, and Cephalosporin (**Table 1**). The haloarchaea were also resistant to Ampicillin (ß-lactam), Tetracycline, Erythromycin, Chloramphenicol (Phenicols), and Clindamycin (Lincosamide). But these antibiotic are known to have no anti-archaeal activity due to unique structural and biochemical properties of archaea (Khelaifia and Drancourt, 2012). Antibiotics inhibiting cell wall and peptidoglycan synthesis viz. ß-Lactams, glycopeptides, lipoglycopeptide, and fosfomycin have no anti-archaeal activity (Khelaifia and Drancourt, 2012) as the cell wall of haloarchaea lack peptidoglycan and is constituted of S-layer and pseudomureins. Archaea also demonstrate differential response to protein synthesis inhibitors and appear to be resistant to antibiotics like macrolides, tetracyclines, erythromycin, and lincosamides that target 30S or 50S ribosomal subunit (Xue et al., 2005; Khelaifia and Drancourt, 2012). The basis of archaeal resistance to these protein synthesis inhibitors may be due to the impermeable nature of archaeal membrane that may not allow the antibiotic to be transported inside the cell or due to the absence of a ribosomal binding site (Elhardt and Böck, 1982). Similarly, archaea are resistant to cell wall altering antibiotic like polymyxin. *Halalkalicoccus tibetensis* has been reported to be resistant to penicillin, ampicillin, polymyxin, streptomycin, tetracycline, bacitracin, neomycin, and sulphafurazole (Xue et al., 2005). The antibiotics inhibiting DNA synthesis and transcription are more effective against archaea. Khelaifia and Drancourt (2012) have reported anti-archaeal activity by DNA interfering antimicrobials namely Novobiocin, Imidazole, Nitrofurans, Sulphonamides, and Benzylpyrimidines. MDR bacteria are defined as non-susceptibility to at least one agent in three or more antimicrobial categories while XDR bacteria are non-susceptible to at least one agent in all but two or fewer antimicrobial categories (Magiorakos et al., 2012). Currently the data regarding drug resistance and break points as per Clinical and Laboratory Standards Institute (CLSI) for classification of multi drug resistance in haloarchaea is unavailable. Nevertheless, as per CLSI and EUCAST and on the basis of the definition described for MDR-bacteria (Magiorakos et al., 2012; Mapara et al., 2015), the haloarchaea used in the present study can be referred as MDR-haloarchaea due to its non-susceptibility to at least one agent in three or more antimicrobial categories which are particularly specified for its anti-archaeal inhibition (Quinolones, Aminoglycosides, Cephalosporins; Khelaifia and Drancourt, 2012). However, owing to the basic differences in cell structure between bacteria and archaea, there is an impeding need for guidelines to be prescribed exclusively for haloarchaea suggesting the use of appropriate antibiotics, their dose. MIC and break points for describing the multi drug resistance in haloarchaea.

The ecological habitats of haloarchaea act like metal sincs and traps for accumulation of metal ions (Chapman and Wang, 2001). When haloarchaea are exposed to heavy metals like Ag, Co, Ni, Cd, Cr, Hg, Pb, Cu, and Zn in their natural habitats, they adapt and survive the metal toxicity and maintain their cellular homeostasis by the activation of molecular mechanisms of metal resistance like the upregulation of P_1B_-type ATPases, ABC transporters, metallochaperones and cation diffusion facilitators (Srivastava and Kowshik, 2013). There is no doubt that these haloarchaea are indomitable extremophiles and are capable of survival in environments that challenge organismic growth. However, studies related to the survival and responses of these extreme haloarchaea to nanoparticles are still largely unexplored. Salgaonkar et al. (2016) have reported the resistance of haloarchaea to ZnO nanoparticles, however, there are no reports on effect of SNPs on these extremophiles. The present study was an attempt to assess the response of haloarchaea to biosynthesized SNPs and study the possible mechanism of the toxicity of SNPs on the viability of these extremophiles.

The SNPs used in this study were synthesized using the dried leaves of *Cinnamomum tamala* known as Indian bay leaf or Malabar leaf. *C. tamala* is a common, easily available edible spice used for culinary purposes as a condiment in Indian and Asian cuisine. There are many advantages of using *C. tamala* leaves for synthesis of the SNPs. The plant material is rich in phytoconstituents and is easily available through-out the year, the cost of the material is economical, the plant material is edible and is non-toxic to human cells. Hence, this spice was preferred for synthesis of SNPs instead of harnessing medicinally important and rare seasonal plants that occur in forest areas or specialized eco-niches. The complex corona that surrounds the nanoparticles plays a significant role in determining the size, shape, composition, and application of the nanomaterial (Daima et al., 2013). The toxicity of the biosynthesized SNP also depends on the nature of the plant material used for biogenesis and is enhanced if the biostabilization or functional capping of the SNPs is by bioactives, plant peptides, and phytoconstituents. The size of the spherical SNPs was between 25 and 50 nm as characterized by SEM and XRD, they were crystalline in nature as evidenced by XRD pattern. The plant material *C. tamala* used in this study is known to contain bioactives like pinene, sesquiterpenes, phellandrenes, geraniol, linalool, and phenolics (Devi et al., 2007) which may be advantageous in enhancing the toxicity of SNPs produced by *C. tamala* makes them potent antagonistic agents (Pal et al., 2007). The phytochemicals studies (FTIR and spectrophotometric assays) revealed presence of high concentrations of phenolic and tannins as well as other phytoconstituents (**Supplementary Table S1**) that may play an active role in stabilizing and capping the SNP. The phytoconstituents may also potentially enhance the antibacterial and cytotoxic efficacies of the *C. tamala* SNPs.

The response and growth of extremely resistant archaea in the presence of SNPs is still unknown. Hence, the antibacterial activities of the SNPs were assessed against extremely resistant haloarchaea. The MIC for SNPs was in the range of 300–400 μg/ml for haloarchaea and 100 μg/ml for gram positive and negative bacteria. Potent inhibition of haloarchaeal and bacterial growth by *C. tamala* SNPs was observed. Many mechanisms have been reported to decipher the inhibitory effect of SNPs on microbial cells. It is proposed that the SNPs of size around 20 nm react with sulfur containing residues in membrane leading to inhibition of enzymatic functioning and inactivation of DNA by reacting with phosphate moieties (Gupta and Silver, 1998). The membrane leakage of reducing sugars and proteins caused due to *C. tamala* SNP was assessed to understand the possible effect of SNPs on haloarchaeal membrane. Membrane leakage of sugars was observed with maximum leakage in *Hfx. prahovense* showing that the SNPs indeed were affecting the membrane permeability and integrity of haloarchaea. Prior studies have reported higher membrane leakage of sugars and proteins in bacteria within 3 h as compared to the membrane leakage observed in haloarchaea (Li et al., 2010; Mapara et al., 2015). This may be attributed to the key difference between bacterial and archaeal cell membranes. The archaeal membranes are more robust and highly resistant than bacteria and are marked by the absence of peptidoglycan, presence of S-layers and occurrence of branched isoprenoid lipids connected by ether linkages to glycerol (Oren, 2008, 2012). Inspite of their resilience, the membrane integrity of the haloarchaea was affected by the *C. tamala* SNPs demonstrating its potency. SNPs of size lesser than 20 nm are known to affect the membrane permeability of microorganisms leading to cell death (Matsumura et al., 2003; Morones et al., 2005).

Silver nanoparticles and silver ions are also known to inhibit bacterial growth by uncoupling the electron transport chain of bacteria, inhibit respiratory chain dehydrogenases and disrupt oxidative phosphorylation (Bard and Holt, 2005; Marambio-Jones and Hoek, 2010). The effect of SNPs on destabilization of membrane respiratory chain of haloarchaea was studied by the reduction of INT by the archaeal respiratory chain dehydrogenases. In the present study, the respiratory chain dehydrogenase activity of *Hal. tradensis* and *Hfx. lucentense* showed a time dependent decrease on treatment with SNPs while respiratory activities of *Hal. argentinensis* and *Hfx. prahovense* showed a significant increase. Most bacteria show a decreased respiratory chain dehydrogenase activity when exposed to SNPs (Li et al., 2010; Mapara et al., 2015). The differences in the obtained results is due to the apparent difference in bacterial and archaeal respiratory chain. Halophilic archaea have a slightly modified electron transport chain which is branched and has menaquinones (LoBasso et al., 2008). The respiratory dehydrogenase present in haloarchaea used in this study are dependent on NADH, succinate, and glycerophosphate (GP) and requires NaCl for integrity. The organisms *Hfx. prahovense* used in the current study is also capable of respiration in the presence of arginine or KNO_3_. *Hal. argentinensis* and *Haloferax* sp. have membrane rhodopsin proteins like bacteriorhodopsin or halorhodopsin which are light driven proton pumps (Kandori, 2015). When haloarchaea face oxygen limitation or challenges to respiration, these organisms utilize bacteriorhodopsins to produce proton gradients that generate ATP and allow survival in stress (DasSarma et al., 2012; Thombre et al., 2016a). *Hal. argentinensis* seemed to be the most resistant of the haloarchaea and since it showed no lag phase during growth curve, the increase in dehydrogenase activity after 24 h of treatment of SNP was apparent. Though the respiratory dehydrogenase activity was more than negative control it was significantly lesser than positive control indicating that the electron transport chain was affected by the SNPs.

The damage to haloarchaeal cells by *C. tamala* SNPs was confirmed by studying lipid peroxidation that was detected by the MDA assay. Archaeal lipids are characterized by the presence of isoprenoid side chains linked to an *sn*-glycerol-1-phosphate moiety by an ether linkage (Albers and Meyer, 2011). Though the lipids of archaea differ from bacterial and eukaryotic lipids, MDA is a common indicator of lipid peroxidation even in archaea (Jasso-Chávez et al., 2015). All the four haloarchaea showed increased production of MDA content after 24 h of treatment with SNP. Lipid peroxidation is caused by generation of stress in cells and is associated with production of membrane mediated ROS that causes DNA damage (Perez et al., 2008; Joshi et al., 2011). The peroxidation of lipids due to free radicals and ROS leads to generation of more toxic breakdown products like MDA which was detected in all the four haloarchaea on treatment with SNP.

**FIGURE 7:**
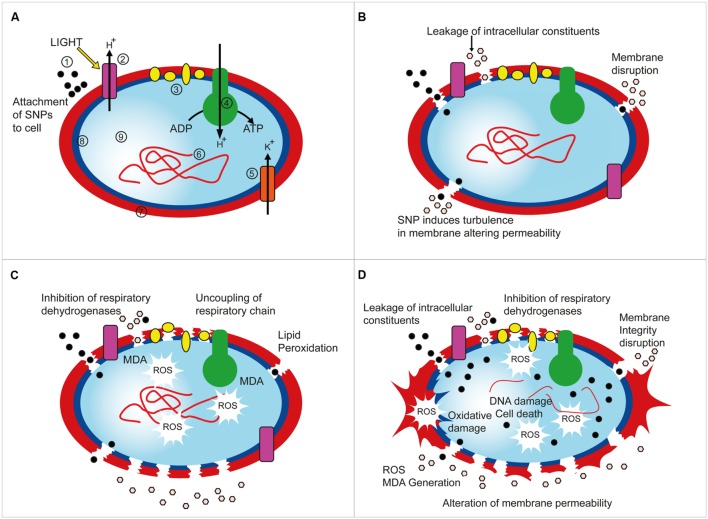
**Proposed mechanism of inhibition of haloarchaea by SNPs.** **(A)** Attachment of SNPs to cell. [1: SNPs, 2: Bacteriorhodopsin protein, 3: Electron transport chain, 4: ATPase complex, 5: Trk type Potassium ion channel, 6: Haloarchaeal genomic DNA, 7: S-layer, 8: Membrane, 9: Intracellular cytosol]. **(B)** Disruption of membrane integrity and leakage of intracellular constituents. **(C)** Inhibition of respiratory dehydrogenases and electron transport chain and generation of MDA and ROS. **(D)** Oxidative, DNA and cellular damage caused by ROS leading to cell death.

The mechanism of inhibition of bacterial growth by SNPs is proposed to be alteration of cell membrane, disruption of electron transport, ROS mediated cellular damage, oxidative damage, and DNA damage (Semeykina and Skulachev, 1990; Marambio-Jones and Hoek, 2010; Nel et al., 2006). SNPs are known to induce apoptosis and inhibit the synthesis on new born DNA in bacteria (Bao et al., 2015). On the basis of the concomitant findings of the present investigation cognate with previous reports on mechanisms of inhibition of SNP against bacteria, we hereby propose a plausible mechanism of action of SNPs against haloarchaea (**Figure 7**). The SNPs may attach to the S-layer proteins, acidic glycoproteins, non-glycosylated proteins by adsorption or other mechanisms (**Figure 7A**). After attachment to the surface of haloarchaea, the SNPs may cause physical damage and form pores in the membrane, disrupt membrane permeability due to turbulence (Mapara et al., 2015), may alter membrane integrity and enter in the cell cytosol leading to leakage of intracellular cytosolic constituents (**Figure 7B**). The SNPs may also be transported through porins or ion channels, however, the mode of internalization of SNPs in cell is largely unclear. While damaging membrane integrity, the SNPs may affect the respiratory dehydrogenases and electron transport chain leading to generation of intracellular stress, lipid peroxidation, generation of MDA and reactive oxygen species (ROS; **Figure 7C**). The ROS generated subsequently may cause oxidative damage, DNA damage and cellular damage and all these processes together lead to overall death of the cell (**Figure 7D**). To summarize, the plausible mechanisms of inhibitory action of SNPs on haloarchaea may be due to archaeal membrane leakage by small size of SNPs affecting the membrane integrity, destabilization of the respiratory chain, and lipid peroxidation that generates ROS and MDA leading to killing of the haloarchaea by oxidative and cellular damage, DNA damage and apoptosis (**Figure 7**). Though more studies are needed to gain a deeper insight of the exact step-wise mechanism of SNPs against haloarchaea, this proposed mechanism based on experimental results and prior studies conducted on bacteria may provide a useful outline and direction for future studies on mechanism of action of SNPs on cells. There are many studies conducted on the mechanism of resistance of haloarchaea to stress and metal stress in particular (Srivastava and Kowshik, 2013). However, no studies have been conducted previously to elucidate the causal effects of sensitivity or inhibition of haloarchaea by metallic nanoparticles. This is the first report on an attempt to study the inhibitory activity of SNPs on haloarchaea and decipher the plausible mechanisms of the same. This study may open a new paradigm for further studies on the bioenergetics, behavior of respiratory chain components, elicitation of apoptosis and DNA synthesis inhibition in archaea and other microorganisms caused by the inhibitory action of nanoparticles.

The cytotoxicity of SNP against human cancerous cell lines was studied and an IC-50 value for the *C. tamala* SNPs against A540 and MCF-7 cell lines are 50 and 100 μg/ml, respectively. The biosynthesized SNPs are likely to be capped with functional groups of plant phytochemicals like phenolics, tannins, and flavonoids. These phytochemicals were detected in the plant extracts of *C. tamala* used in the present study (**Figure 1D**). It is reported that SNPs synthesized using plant materials have cytotoxic activity by inhibition of cell growth and mitogen-activated protein kinase pathways. The anti-proliferative activity of SNPs on cancer cell lines is attributed to interference in gene expression, mevalonate depletion, inhibition of HMG-CoA reductase activity, oxidative stress leading to cell cycle inhibition and apoptosis (Duncan et al., 2004; Firdhouse and Lalitha, 2015).

In the current investigation, an environment friendly, facile green synthesis method was adopted for the biogenesis of bio-stabilized SNPs using *C. tamala* leaf extract. The SNPs demonstrated antimicrobial activity against antibiotic resistant haloarchaea. The SNPs also demonstrated potent cytotoxic activities. The plausible mechanism of inhibition of archaeal growth by the SNPs is attributed to the disruption and destabilization of the extremely resistant haloarchaeal membrane and uncoupling of respiratory dehydrogenases along with lipid peroxidation.

## Author Contributions

RT was Principal investigator of project, designed the concept and experiments, did the enrichment of haloarchaea, analyzed the data, drafted the manuscript, and illustrated the diagrams of hypothesis. VS performed experiments related to effect of silver nanoparticles (SNP) on haloarchaea and studied the growth kinetics. ET did the antimicrobial and anticancer activity of SNP, characterized the SNP using zeta potential, FTIR and EDS. SZ synthesized the SNP and studied the antimicrobial activity of SNP. SM performed the XRD and FEG-SEM of SNP.

## Conflict of Interest Statement

The authors declare that the research was conducted in the absence of any commercial or financial relationships that could be construed as a potential conflict of interest.
