# Supplementary material for: Antimicrobial Activity and Mechanism of Inhibition of Silver Nanoparticles against Extreme Halophilic Archaea
Source: Front Microbiol. 2016 Sep 13;7:1424. doi: 10.3389/fmicb.2016.01424 (PMC5020055; doi:10.3389/fmicb.2016.01424)
Supplement: Supplementary file 1 [file Data_Sheet_1.DOCX]

**Antimicrobial Activity and Mechanism of inhibition of Silver Nanoparticles against Extreme Halophilic Archaea**

Rebecca Thombre^1*^, Vinaya Shinde^1^, Elvina Thaiparambil ^1,^ Samruddhi Zende^1^ and Sourabh Mehta^2^

^1^ *Department of Biotechnology, Modern College of Arts, Science and Commerce, Shivajinagar, Pune-411005. Maharashtra, India*

^2^ National center for Nanosciences and Nanotechnology, University of Mumbai, Vidyanagari, Kalina, Santacruz (E), Mumbai -400 098.India.

***Corresponding author:

Dr. Rebecca S. Thombre,

Department of Biotechnology,

Modern College affiliated to S.P. Pune University, Shivajinagar, Pune, 411005.

Email: rebecca.thombre@gmail.com,

Phone no: +91-8390053805

Fax: + (91)-20-25536075

**Supplementary Figures**

**Fig. S1**: Zeta potential of silver nanoparticles synthesized using leaf extract of *C. tamala*

Fig. S2: X-ray diffraction(XRD) pattern of SNPs produced using leaf extract of *C. tamala*

**Supplementary Tables**

**Table S1.** Detection of phytoconstituents present in *C. tamala* leaf extract used for SNP synthesis

**Fig. S1**

|  |  |  |  |  |  |  |  |
| --- | --- | --- | --- | --- | --- | --- | --- |
|  |  |  | **Mean (mV)** | | **Area (%)** | | **Width (mV)** |
| **Zeta Potential (mV):** | -27.3 | **Peak 1:** | -27.3 |  | 100.0 |  | 5.14 |
| **Zeta Deviation (mV):** | 5.14 | **Peak 2:** | 0.00 |  | 0.0 |  | 0.00 |
| **Conductivity (mS/cm):** | 0.785 | **Peak 3:** | 0.00 |  | 0.0 |  | 0.00 |
| **Result quality : Good** | |  |  |  |  |  |  |


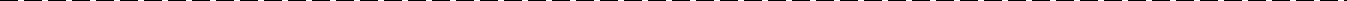

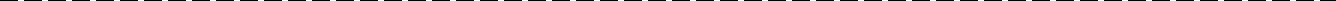


**
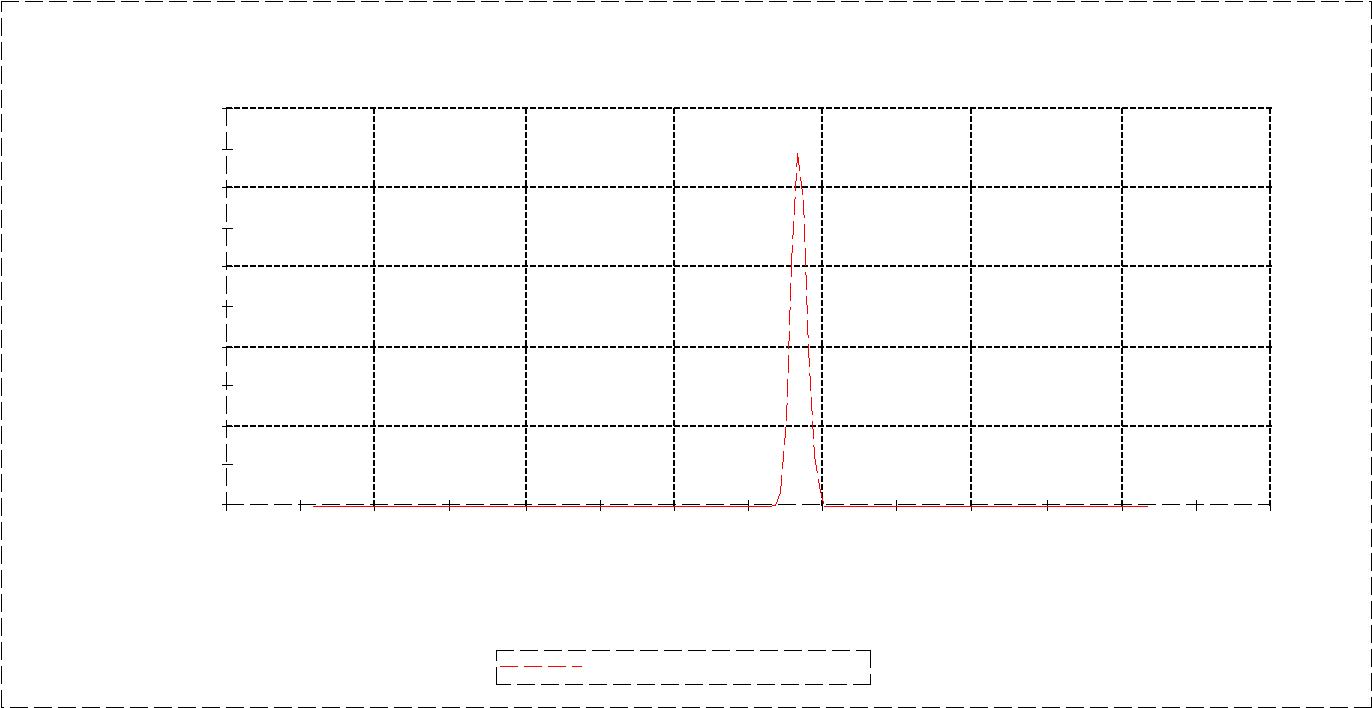
Zeta Potential Distribution**

| *Total Counts* |
| --- |

500000

400000

300000

200000

100000

| 0 |  |  |  |  |  |  |  |
| --- | --- | --- | --- | --- | --- | --- | --- |
| **-400** | **-300** | **-200** | **-100** | **0** | **100** | **200** | **300** |

**Zeta Potential (mV)**

**Fig. S2**

**Table. S1**

| **No** | **Phytoconstituents** | **Estimation method** | **Aqueous extract** | **Ethanolic extract** | **Methanolic extract** |
| --- | --- | --- | --- | --- | --- |
| ^1^ | Alkaloids | Mayer’s test | ^+^ | ^+^ | ^+^ |
| ^2^ | Tannins | Ferric chloride test | ^+^ | ^-^ | ^-^ |
| ^3^ | Flavonoids | Alkaline reagent test | ^+^ | ^+^ | ^-^ |
| ^4^ | Glycosides | Liebermann’s test | ^+^ | ^+^ | ^+^ |
| ^5^ | Saponins | Foam test | ^+^ | ^-^ | ^-^ |
| ^6^ | Carbohydrates | Fehling’s test | ^+^ | ^+^ | ^+^ |
| ^7^ | Protein | Ninhydrin test | ^+^ | ^+^ | ^+^ |
| ^8^ | Antioxidants | DPPH radical Scavenging Assay | ^+^ | ^nd^ | ^nd^ |
| ^9^ | Phenolics^*^ | Folin-Ciocalteau method | 0.232 mg/ml | ^nd^ | ^nd^ |
| ^10^ | Flavonoids^*^ | Aluminum chloride colorimetric method | 0.067mg/ml | ^nd^ | ^nd^ |

Legend: + Present, - Absent, nd – not determined. ^*^Quantitative estimation
